# Supplementary figures and images for: Systematic identification of cancer-associated-fibroblast-derived genes in patients with colorectal cancer based on single-cell sequencing and transcriptomics
Source: Front Immunol. 2022 Aug 29;13:988246. doi: 10.3389/fimmu.2022.988246 (PMC9465173; doi:10.3389/fimmu.2022.988246)

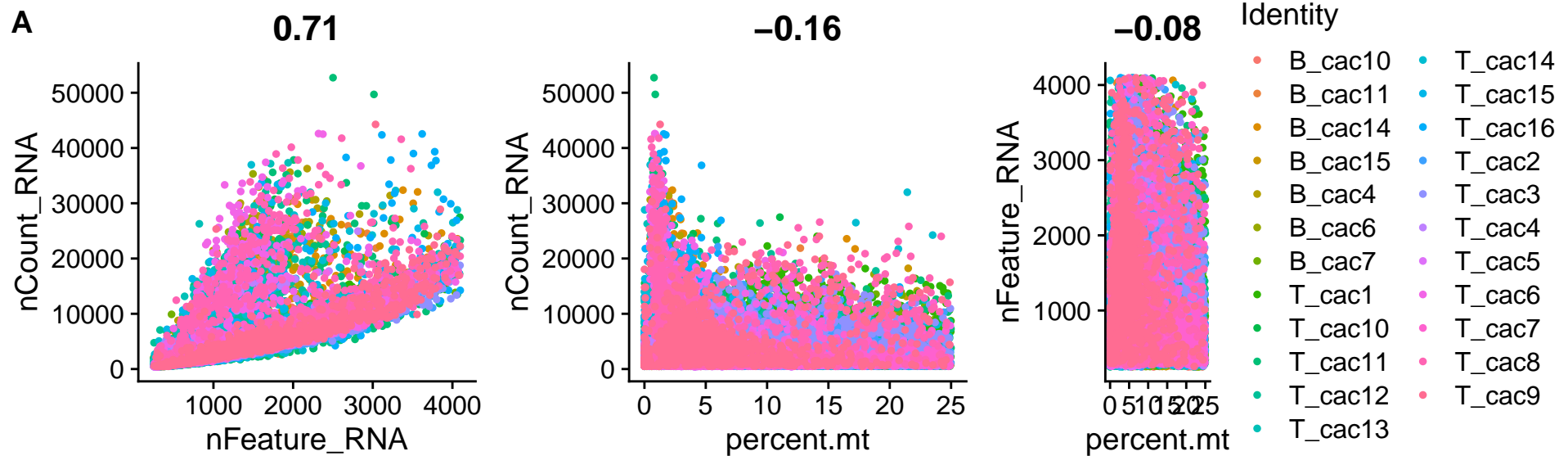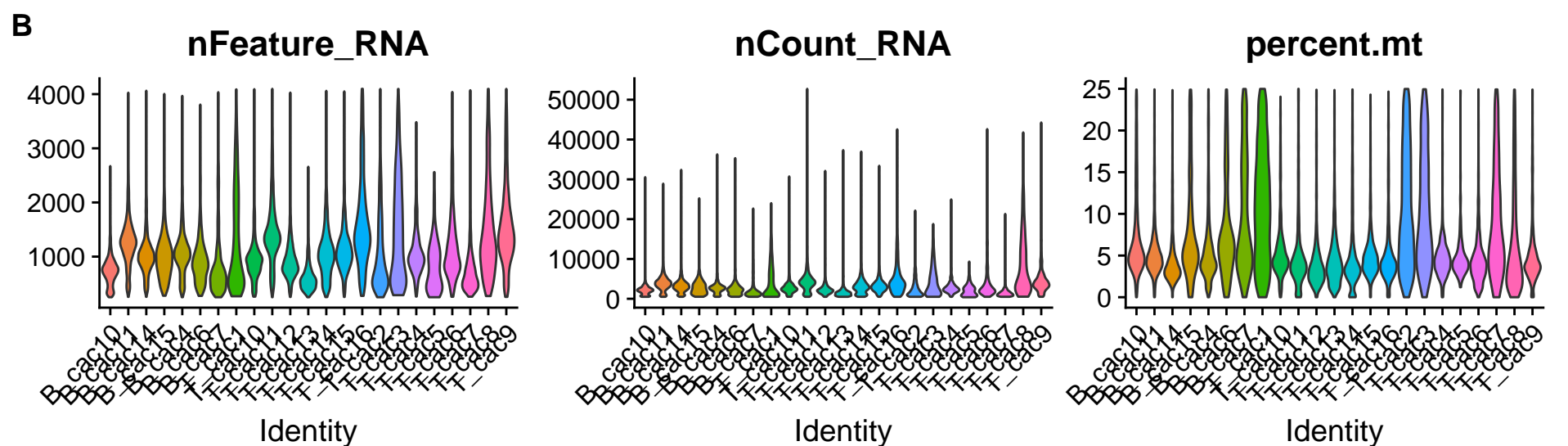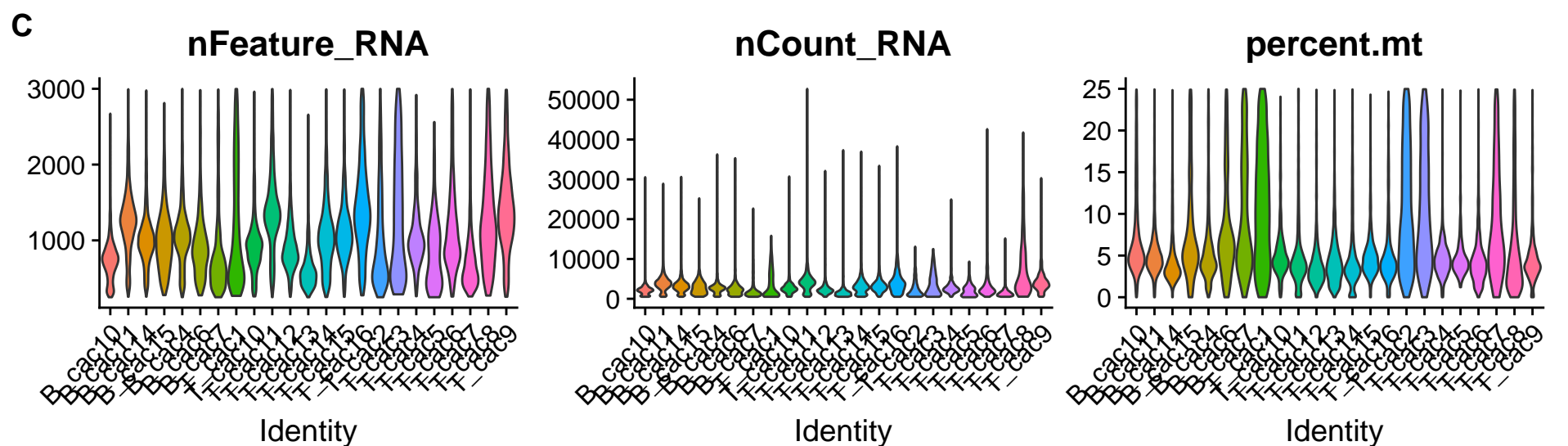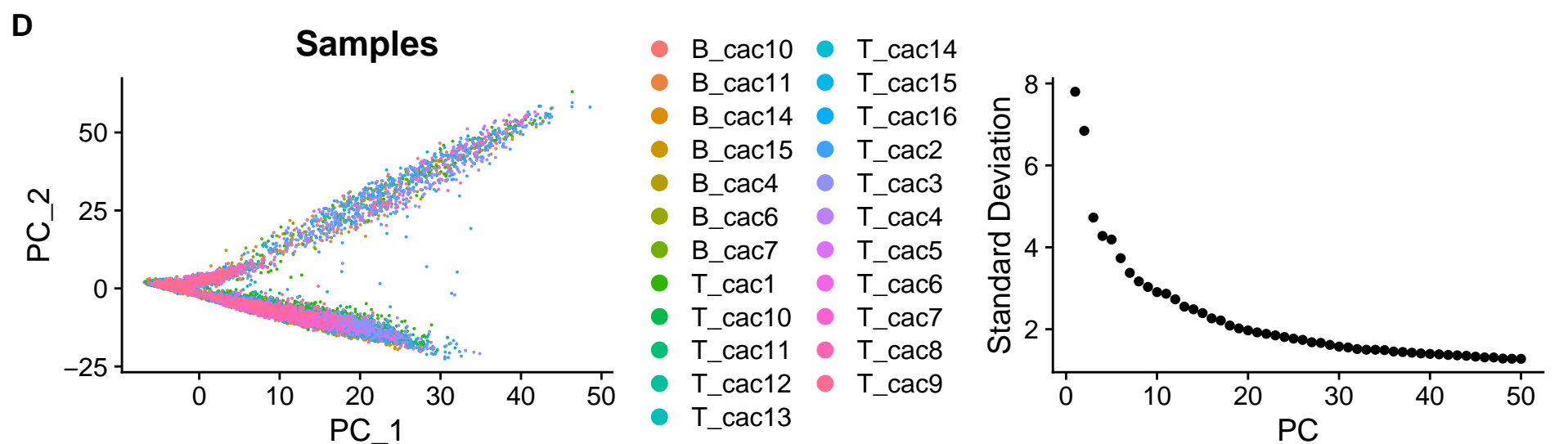

Supplement: Supplementary Figure 1 — (A) Correlation between mitochondrial genes and UMI/mRNA quantity as well as between UMI and mRNA quantity; (B) Correlation among the mRNA/UMI/mitochondrial content/rRNA content of samples before filtration; (C) Correlation among the mRNA/UMI/mitochondrial content/rRNA content of samples after filtration; (D) Dimensionality reduction and identification of anchor points via PCA. [file DataSheet_1.pdf]

**A**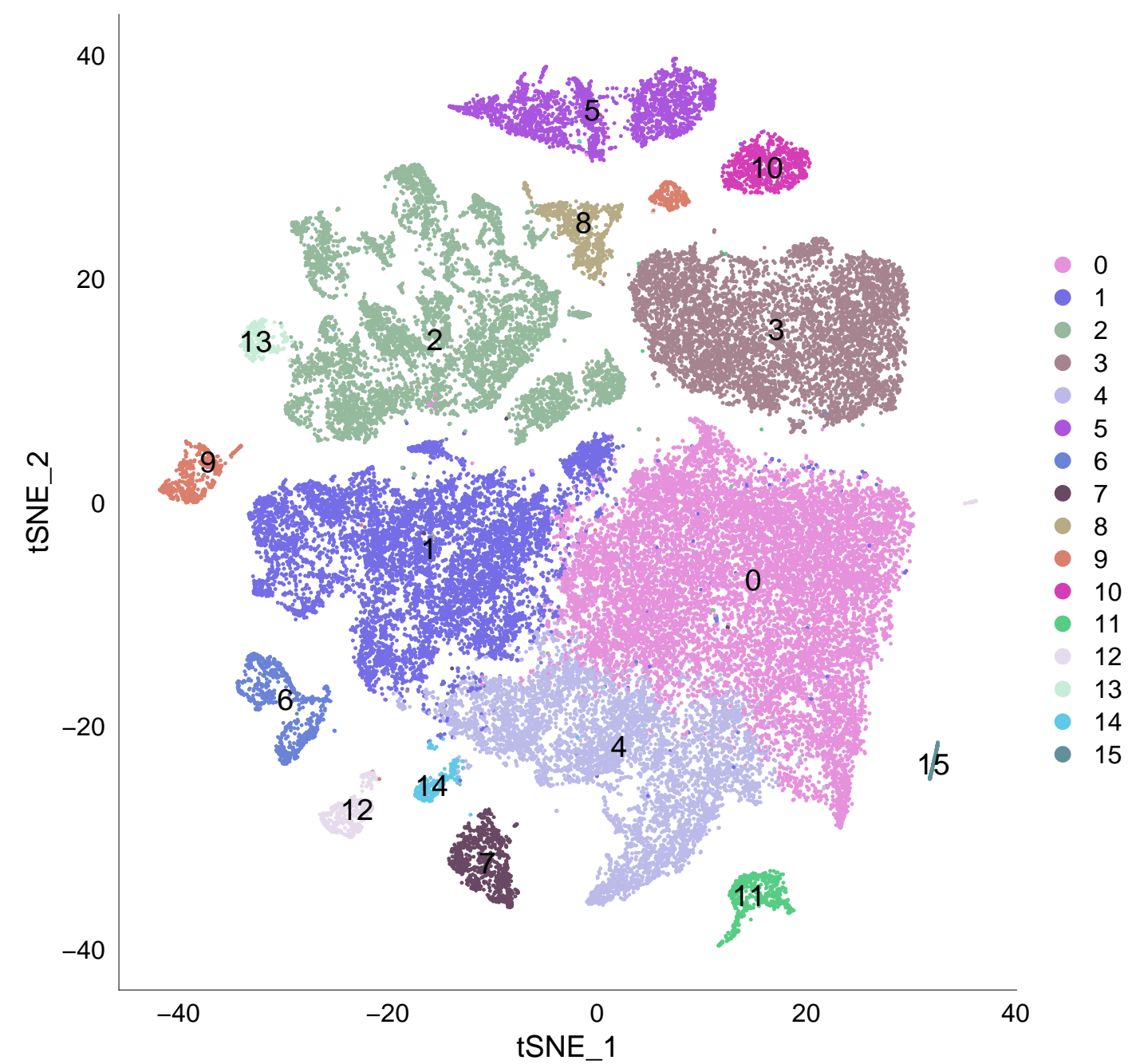**B**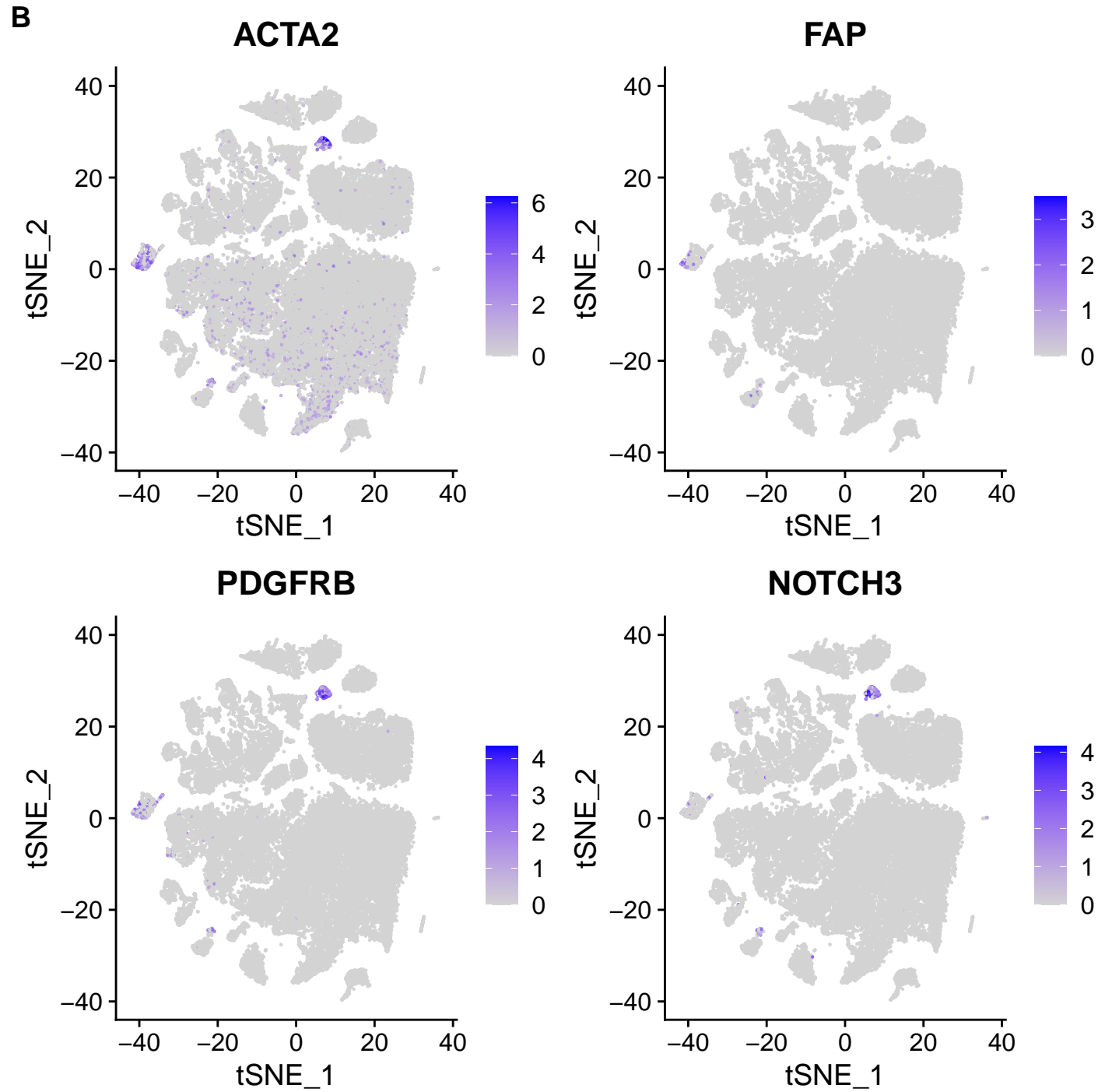**C**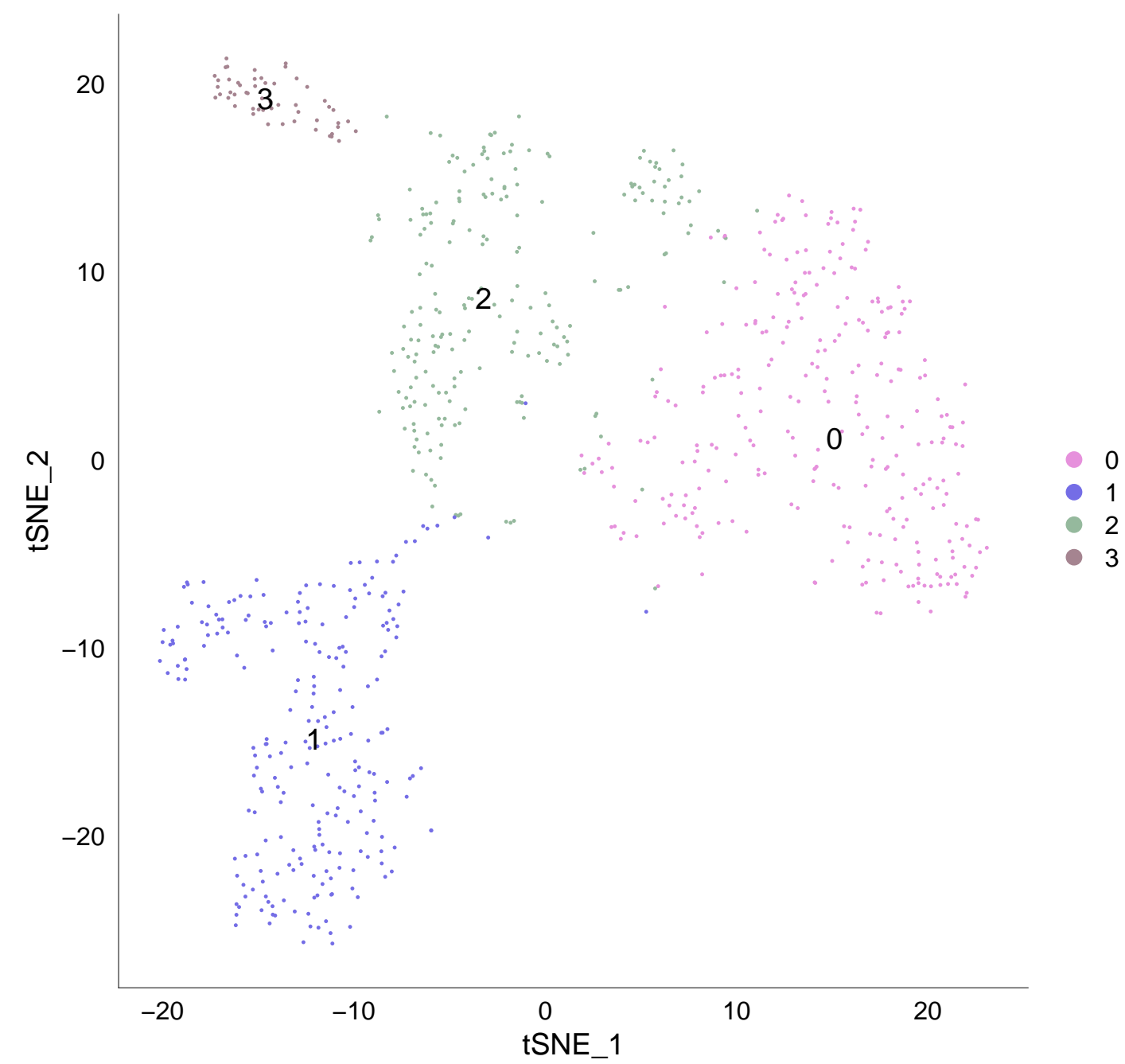**D**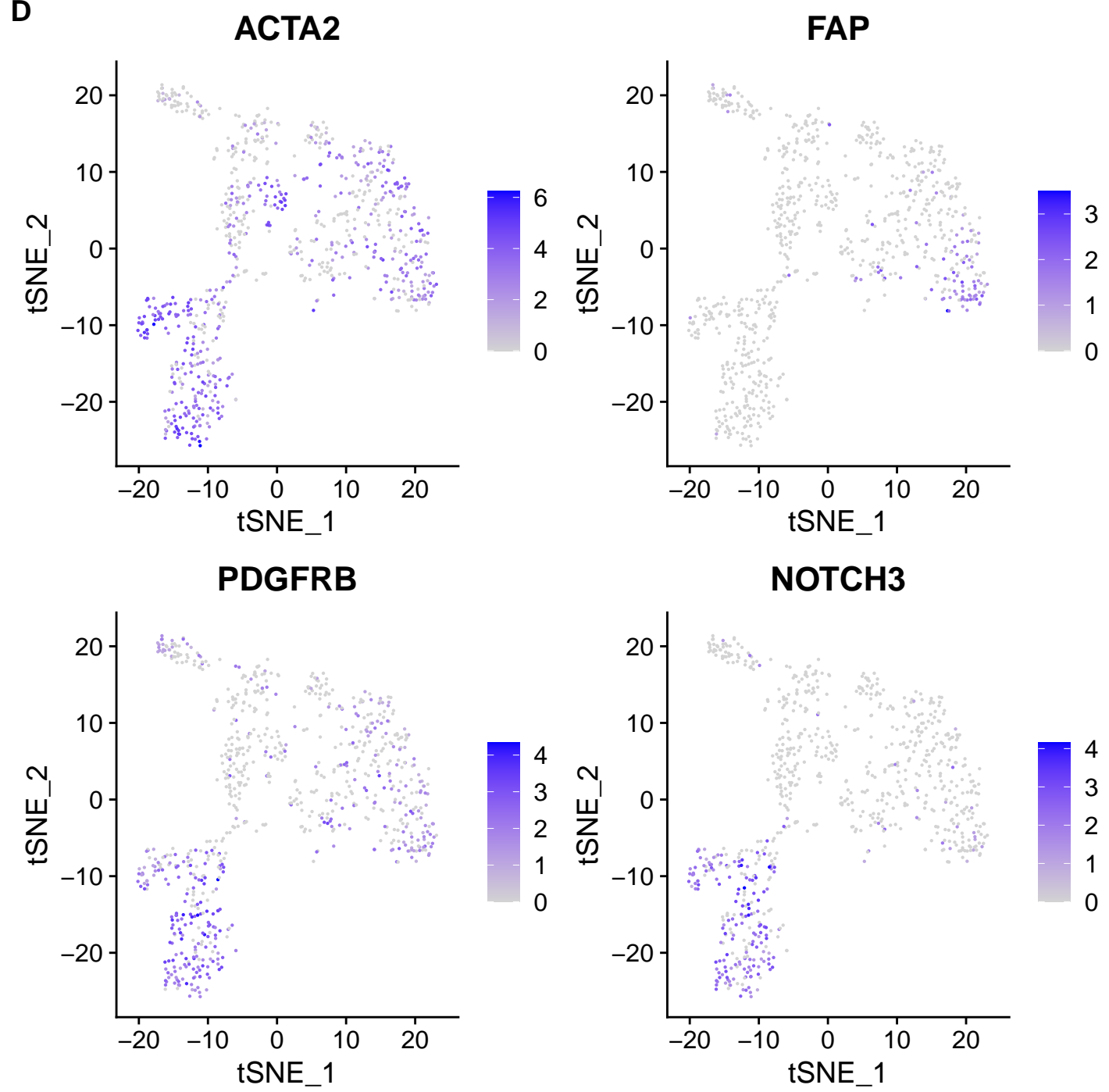

Supplement: Supplementary Figure 2 — (A) Distribution of subpopulations of all cells after cluster analysis; (B) t-SNE map of marker gene expression in fibroblasts; (C) Distribution of fibroblast subgroups after re-clustering; (D) t-SNE map of marker gene expression in four small fibroblast subpopulations. [file DataSheet_2.pdf]
